# Characterization of resistance and virulence factors in livestock-associated methicillin-resistant *Staphylococcus aureus*

**DOI:** 10.1038/s41598-024-63963-3

**Published:** 2024-06-09

**Authors:** Abeni Beshiru, Isoken H. Igbinosa, Olajide Akinnibosun, Abraham G. Ogofure, Afamefuna Dunkwu-Okafor, Kate E. Uwhuba, Etinosa O. Igbinosa

**Affiliations:** 1 Applied Microbial Processes & Environmental Health Research Group, Faculty of Life Sciences, University of Benin, PMB 1154, Benin City, 300283 Nigeria; 2 Department of Microbiology, College of Natural and Applied Sciences, Western Delta University, Oghara, Nigeria; 3 Department of Environmental Management & Toxicology, Faculty of Life Sciences, University of Benin, PMB 1154, Benin City, 300283 Nigeria; 4 Department of Microbiology, Faculty of Science, Federal University of Health Sciences, PMB 145, Otukpo, 927101 Otukpo Nigeria

**Keywords:** Antimicrobial resistance, Microbiology, Biofilms, Cellular microbiology

## Abstract

The study investigated the economic concerns associated with livestock-associated methicillin-resistant *Staphylococcus aureus* (LA-MRSA) in livestock (cow), examining its connection to severe infections, antimicrobial resistance (AMR), and virulence factors. The research, conducted in Edo State, Nigeria, analyzed 400 samples (200 rectal and 200 nasal swabs) collected between March 2018 and February 2019. MRSA prevalence was identified using conventional culture-based methods and polymerase chain reaction (PCR) techniques, revealing 63.5% (n = 254) for *Staphylococcus aureus* and 55% (n = 220) for MRSA. Of the 76 *mecA*-positive MRSA isolates, 64.5% (n = 49) exhibited multidrug resistance (MDR) while the remaining were sensitive to specific antimicrobials. Key virulence genes, such as *PVL* (81.6%; n = 62) and *tsst-1* (44.7%; n = 34), were prevalent, along with AMR genes like *mecC*, *tetM*, *ermA*, *ermC*, *vanA*, and *vanC*. Staphylococcal chromosomal cassette mec (*SCCmec*) typing identified different types, notably II, IVa, and IVb. Biofilm formation, a crucial virulence factor varied in strength, is associated with *icaA* and *icaB* genes (*p* < 0.01). The findings highlighted substantial AMR and biofilm-forming capacity within LA-MRSA isolates, emphasizing the importance of ongoing surveillance for informed treatment strategies, AMR policies, and control measures against MDR staphylococcal infections.

## Introduction

*Staphylococcus aureus* (*S. aureus*) is a key pathogen responsible for severe infections, including osteomyelitis, pneumonia, and endocarditis, leading to conditions like sepsis and bacteremia^1^. Methicillin-resistant *S. aureus* (MRSA) exhibits reduced susceptibility to beta-lactam antibiotics, attributed to the replacement of antibiotic-sensitive penicillin-binding proteins (PBPs) with the acquired PBP2a^2^. This resistance mechanism is governed by staphylococcal chromosomal cassettes (*SCCmec*), housing *mecA* or *mecC* genes^3^. *SCCmec* subtypes I-V contribute significantly to antimicrobial resistance (AMR), harbouring genetic determinants for resistance to tetracyclines, beta-lactams, aminoglycosides, and macrolides, resulting in the rise of multidrug-resistant (MDR) MRSA^4^. MRSA, causing around 150,000 annual cases in Europe and 7,000 deaths, includes livestock-associated strains (LA-MRSA) found in cattle, pigs, and poultry, posing a zoonotic threat^5^. This transmission risk is prominent among individuals with substantial exposure, such as veterinarians and farmers^6^. Facilitation of the transfer of resistance genes occurs through horizontal gene transfer by the presence of AMR genes within mobile genetic elements (MGEs) like *SCCmec*, emphasizing the need to understand these molecular characteristics for effective control measures.

In the realm of LA-MRSA, the ST398 sequence type is frequently associated with human infections, highlighting a potential zoonotic transmission risk^7^. LA-MRSA demonstrates the ability to reacquire MRSA genes linked to human invasiveness, showcasing its adaptability across host environments^8^. Virulence factors like toxic shock syndrome toxin (*tsst*) and Panton-Valentine leukocidin (*PVL*) significantly impact MRSA pathogenicity. The tetracycline resistance gene *tetM* is notably prevalent in MDR MRSA strains, adding complexity to antibiotic resistance patterns^9^. Recognizing MRSA as an important pathogen, the World Health Organization (WHO) acknowledges its propensity to acquire AMR determinants, often associated with a diverse array of virulence factors. Crucially, MRSA strains inherently resist all β-lactam therapeutic agents, including carbapenems and cephalosporins^10^. The predominant antimicrobial agents administered for *S. aureus* infections contribute to the rising occurrence of AMR-related cases, emphasizing the consequences of misuse and unrestricted use in livestock farming^4^.

The imprudent usage of antimicrobial agents has fueled selective pressure, driving the surge in AMR, particularly in *S. aureus*, giving rise to MDR strains. Colonized mammals, including those in Nigeria, serve as reservoirs for both methicillin-susceptible *S. aureus* and MRSA^11^. MRSA hasnt been identified across diverse sources, including sheep, humans, slaughtered animals, raw milk, poultry, and more^2,12–15^. Notably, a substantial gap in knowledge exists regarding the molecular characteristics of MRSA in Nigerian cows. Research indicates that animal-derived MRSA strains exhibit heightened resistance to various antibiotics compared to human-derived strains^3^, emphasizing the need for targeted understanding and intervention. Widespread usage of antimicrobials as preventive measures and growth promoters in the livestock industry has surged, with 131,109 tons of antibiotics used in 2013, projected to exceed 200,000 tons annually by 2030^16^. This extensive use raises concerns about antibiotic resistance, potentially causing 10 million annual fatalities by 2050^17^. Inefficient antibiotic absorption in livestock leads to the excretion of residues in biological waste, accumulating over time. This reckless antibiotic use contributes to a heightened prevalence of antibiotic-resistant bacteria in livestock settings, posing a significant risk to other ecosystems, including the human food chain^18^.

The surge in AMR bacteria led the European Union (EU) to ban antibiotic use as growth promoters in 2006. Nigeria's Ministry of Agriculture advises against antibiotics for enhanced egg production and animal growth, urging strict adherence to veterinarian prescriptions^19^. Despite recommendations, routine antibiotic use persists in veterinary practices for prophylaxis and infection treatment. In 2015, the EU reported 8361 tons of veterinary antimicrobial use; tetracycline and penicillin are the most commonly prescribed antibiotics for animals in food production^20^. This aligns with World Antimicrobial Awareness Week, which aims to raise awareness among stakeholders about practices to curb drug-resistant microorganisms. In Nigeria, lax enforcement allows livestock farmers unrestricted antibiotic access, contributing to indiscriminate purchases and fostering self-medication issues in human medicine^19^. *S. aureus* possesses the crucial ability to form biofilms, boosting resistance to microbial control in food-producing animals^2^. Biofilm activities involving Microbial Surface Components Recognizing Adhesive Matrix Molecules (MSCRAMMs) and regulated by the *icaABCD* operon contribute to the prevalence of *S. aureus* in animal-derived products^3^. While research on LA-MRSA and occupational risks is extensive^9^, Nigeria needs more comprehensive insights into its molecular traits. This study in Edo, Nigeria, delves into LA-MRSA's role in MRSA epidemiology in cows, characterizing strains through antimicrobial testing, genetic element examination, and virulence profiling.

## Results

### Prevalence of *S. aureus* and MRSA from food-producing animals (cows)

From the 400 samples (200 nasal and rectal specimens) obtained from the same animal source, 254(63.5%) were positive for *S. aureus*, while 220(55%) were positive for MRSA based on cultural and biochemical characteristics and molecular detection of specific marker genes. From the 200 rectal specimens, 143(71.5%) were positive for *S. aureus*, while 122(61%) were positive for MRSA. From the 200 nasal specimens, 111(55.5%) were positive for *S. aureus*, while 98(49%) were positive for MRSA (Table 1). A total of 76 MRSA isolates (38 nasal and 38 rectal) were selected and characterized further. All 76 isolates also harboured the *mecA* gene for MRSA confirmation. The methicillin-sensitive *S. aureus* was not characterized further.

**Table 1:** Occurrence of *S. aureus* from free-range cows.

The number of samples collected	Number of positive samples using BPA	Number of positive samples using MRSA agar
Rectal-200	143 (71.5)	122 (61)
Nasal-200	111 (55.5)	98 (49)
Total-400	254 (63.5)	220 (55)

Values in parenthesis represent percentage (%); *BPA* Baird-Parker agar, *MRSA* Methicillin-resistant *S. aureus.*

### Antimicrobial susceptibility profile of the MRSA isolates

All 38 (100%) nasal and rectal isolates exhibited resistance to penicillin, ceftaroline, and cefotaxime (Table 2). Nasal cavity isolates additionally displayed resistance to ertapenem 21 (55.3%), clindamycin 19 (50%), tetracycline 16 (42.1%), meropenem 14 (36.8%), piperacillin 10 (26.3%), erythromycin 10 (26.3%), ciprofloxacin 10 (26.3%), vancomycin 9 (23.7%), and kanamycin 5 (13.2%). Rectal cavity isolates also demonstrated resistance to ertapenem 30 (78.9%), clindamycin 24 (63.2%), tetracycline 20 (52.6%), meropenem 21 (55.3%), piperacillin 17 (44.7%), erythromycin 17 (44.7%), ciprofloxacin 15 (39.5%), vancomycin 17 (44.7%), and kanamycin 11 (28.9%). Nasal isolates exhibited sensitivity to kanamycin 30 (78.9%), gentamicin, and vancomycin 25 (65.8%). Rectal isolates demonstrated sensitivity to kanamycin 19 (50%), gentamicin, and vancomycin 14 (36.8%). All isolates 76 (100%) exhibited susceptibility to linezolid, quinupristin-dalfopristin, oritavancin, teicoplanin, daptomycin, and tedizolid.

**Table 2:** Antimicrobial susceptibility of MRSA from free-range cows.

Antimicrobial class	Antibiotics	Nasal isolates (*n* = 38)			Rectal isolates (*n* = 38)			Total isolates (*n* = 76)		
		R	I	S	R	I	S	R	I	S
Penicillins	PEN	38 (100)	0 (0)	0 (0)	38 (100)	0 (0)	0 (0)	76 (100)	0 (0)	0 (0)
	PTZ	10 (26.3)	17 (44.7)	11 (28.9)	17 (44.7)	19 (50)	2 (5.3)	27 (35.5)	36 (47.4)	13 (17.1)
Streptogramins	QDA	0 (0)	0 (0)	38 (100)	0 (0)	0 (0)	38 (100)	0 (0)	0 (0)	76 (100)
Carbapenems	IMI	3 (7.9)	21 (55.3)	14 (36.8)	5 (13.2)	23 (60.5)	10 (26.3)	8 (10.5)	44 (57.9)	24 (31.6)
	ETP	21 (55.3)	11 (28.9)	6 (15.8)	30 (78.9)	8 (21.1)	0 (0)	51 (67.1)	19 (25)	6 (7.9)
	MEM	14 (36.8)	19 (50)	5 (13.2)	21 (55.3)	13 (34.2)	4 (10.5)	35 (46.1)	32 (42.1)	9 (11.8)
Lipoglycopeptides	ORI	0 (0)	0 (0)	38 (100)	0 (0)	0 (0)	38 (100)	0 (0)	0 (0)	76 (100)
	TEI	0 (0)	0 (0)	38 (100)	0 (0)	0 (0)	38 (100)	0 (0)	0 (0)	76 (100)
Aminoglycosides	KAN	5 (13.2)	3 (7.9)	30 (78.9)	11 (28.9)	8 (21.1)	19 (50)	16 (21.1)	11 (14.5)	49 (64.5)
	GEN	7 (18.4)	6 (15.8)	25 (65.8)	13 (34.2)	11 (28.9)	14 (36.8)	20 (26.3)	17 (22.4)	39 (51.3)
Tetracyclines	TET	16 (42.1)	12 (31.6)	10 (26.3)	20 (52.6)	17 (44.7)	1 (2.6)	36 (47.4)	29 (38.2)	11 (14.5)
Lincosamide	CLI	19 (50)	18 (47.4)	1 (2.6)	24 (63.2)	13 (34.2)	1 (2.6)	43 (56.6)	31 (40.8)	2 (2.6)
Macrolides	ERY	10 (26.3)	17 (44.7)	11 (28.9)	17 (44.7)	18 (47.4)	3 (7.9)	27 (35.5)	35 (46.1)	14 (18.4)
Lipopeptides	DAP	0 (0)	0 (0)	38 (100)	0 (0)	0 (0)	38 (100)	0 (0)	0 (0)	76 (100)
Fluoroquinolone	CIP	10 (26.3)	15 (39.5)	13 (34.2)	15 (39.5)	19 (50)	4 (10.5)	25 (32.9)	34 (44.7)	17 (22.4)
Oxazolidinones	LIZ	0 (0)	9 (23.7)	29 (76.3)	0 (0)	5 (13.2)	33 (86.8)	0 (0)	14 (18.4)	62 (81.6)
	TED	0 (0)	0 (0)	38 (100)	0 (0)	0 (0)	38 (100)	0 (0)	0 (0)	76 (100)
Cephalosporins	CRO	38 (100)	0 (0)	0 (0)	38 (100)	0 (0)	0 (0)	76 (100)	0 (0)	0 (0)
	CTX	38 (100)	0 (0)	0 (0)	38 (100)	0 (0)	0 (0)	76 (100)	0 (0)	0 (0)
Glycopeptides	VAN	9 (23.7)	4 (10.5)	25 (65.8)	17 (44.7)	7 (18.4)	14 (36.8)	26 (34.2)	11 (14.5)	39 (51.3)

*PEN* Penicillin G, *PTZ* Piperacillin, *IMI* Imipenem, *ETP* Ertapenem, *MEM* Meropenem, *KAN* Kanamycin, *GEN* Gentamicin, *TET* Tetracycline, *CLI* Clindamycin, *ERY* Erythromycin, *CIP* Ciprofloxacin, *CRO* Ceftaroline, *CTX* Cefotaxime, *VAN* Vancomycin, *LIZ* Linezolid, *QDA* Quinupristin-dalfopristin, *ORI* Oritavancin, *TEI* Teicoplanin, *DAP* Daptomycin, and *TED* Tedizolid. Values in parentheses represent percentages (%).

### MDR and multiple antibiotic resistance (MAR) index of the MRSA isolates

MDR profile from the nasal cavity reveals that 20(52.6%) were resistant to 4 antibiotics (PEN^R^, ETP^R^, CRO^R^, CTX^R^) in 3 different antimicrobial classes with a MAR index of 0.20. In addition, 8(21.1%) of isolates from the nasal cavity were also resistant to 11 antibiotics (PEN^R^, PTZ^R^, ETP^R^, MEM^R^, TET^R^, CLI^R^, ERY^R^, CIP^R^, CRO^R^, CTX^R^, VAN^R^) from 8 different class of antibiotics with a MAR index of 0.55. MDR profile from the rectal cavity reveals that 29(76.3%) were resistant to 4 antibiotics (PEN^R^, ETP^R^, CRO^R^, CTX^R^) in 3 different antimicrobial classes with a MAR index of 0.20. In addition, 10(26.3%) of isolates from the rectal cavity were also resistant to 13 antibiotics (PEN^R^, PTZ^R^, ETP^R^, MEM^R^, KAN^R^, GEN^R^, TET^R^, CLI^R^, ERY^R^, CIP^R^, CRO^R^, CTX^R^, VAN^R^) from 9 different class of antibiotics with a MAR index of 0.65 (Table 3). Overall, 49(64.5%) of the MRSA isolates were MDR. None of the investigated were pan-drug resistant or extensively drug-resistant.

**Table 3:** MDR and MAR index of the MRSA isolates.

Isolates	No antimicrobial class	No of antibiotics	Resistance phenotype	No resistant isolates	MAR index
Nasal isolates (*n* = 38)	3	4	PEN^R^, ETP^R^, CRO^R^, CTX^R^	20 (52.6)	0.20
	5	6	PEN^R^, ETP^R^, CLI^R^, TET^R^, CRO^R^, CTX^R^	15 (39.5)	0.30
	5	7	PEN^R^, ETP^R^, MEM^R^, CLI^R^, TET^R^, CRO^R^, CTX^R^	13 (34.2)	0.35
	8	11	PEN^R^, PTZ^R^, ETP^R^, MEM^R^, TET^R^, CLI^R^, ERY^R^, CIP^R^, CRO^R^, CTX^R^, VAN^R^	8 (21.1)	0.55
Rectal isolates (*n* = 38)	3	4	PEN^R^, ETP^R^, CRO^R^, CTX^R^	29 (76.3)	0.20
	4	5	PEN^R^, ETP^R^, CLI^R^, CRO^R^, CTX^R^	22 (57.9)	0.25
	5	7	PEN^R^, ETP^R^, MEM^R^, TET^R^, CLI^R^, CRO^R^, CTX^R^	19 (50.0)	0.35
	7	10	PEN^R^, PTZ^R^, ETP^R^, MEM^R^, TET^R^, CLI^R^, ERY^R^, CRO^R^, CTX^R^, VAN^R^	16 (42.1)	0.50
	9	13	PEN^R^, PTZ^R^, ETP^R^, MEM^R^, KAN^R^, GEN^R^, TET^R^, CLI^R^, ERY^R^, CIP^R^, CRO^R^, CTX^R^, VAN^R^	10 (26.3)	0.65
Total isolates (*n* = 76)	3	4	PEN^R^, ETP^R^, CRO^R^, CTX^R^	49 (64.5)	0.20
	4	5	PEN^R^, ETP^R^, CLI^R^, CRO^R^, CTX^R^	41 (53.9)	0.25
	5	7	PEN^R^, ETP^R^, MEM^R^, TET^R^, CLI^R^, CRO^R^, CTX^R^	33 (43.4)	0.35
	8	11	PEN^R^, PTZ^R^, ETP^R^, MEM^R^, TET^R^, CLI^R^, ERY^R^, CIP^R^, CRO^R^, CTX^R^, VAN^R^	22 (28.9)	0.55
	9	14	PEN^R^, PTZ^R^, IMI^R^, ETP^R^, MEM^R^, KAN^R^, GEN^R^, TET^R^, CLI^R^, ERY^R^, CIP^R^, CRO^R^, CTX^R^, VAN^R^	7 (9.2)	0.70

*PEN* Penicillin G, *PTZ* Piperacillin, *IMI* Imipenem, *ETP* Ertapenem, *MEM* Meropenem, *KAN* Kanamycin, *GEN* Gentamicin, *TET* Tetracycline, *CLI* Clindamycin, *ERY* Erythromycin, *CIP* Ciprofloxacin, *CRO* Ceftaroline, *CTX* Cefotaxime, *VAN* Vancomycin. Values in parentheses represent percentages (%).

### AMR and virulence determinants of the MRSA isolates

**Figure 1:**
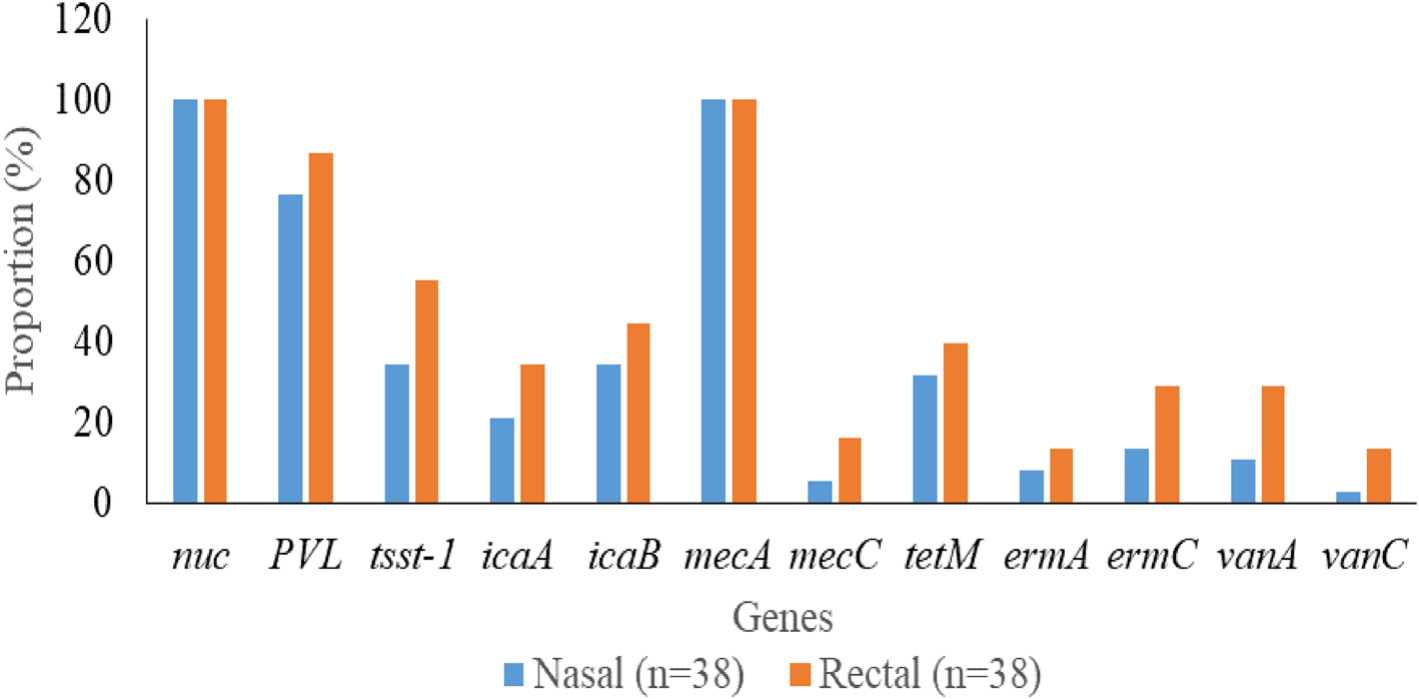
Distribution of resistance and virulence genes in the staphylococcal isolates.

Virulence genes detected include *PVL* 62/76 (81.6%), *nuc* 76/76 (100%), *tsst-1* 34/76 (44.7%), *icaA* 21/76 (27.6%), and *icaB* 30/76 (39.5%). AMR genes detected include *mecA* 76/76 (100%), *mecC* 8/76 (10.5%), *tetM* 27/76 (35.5%), *ermA* 8/76 (10.5%), *ermC* 16/76 (21.1%), *vanA* 15/76 (19.7%), and *vanC* 6/76 (7.9%) (Fig. 1). All isolates harbouring AMR genes exhibited phenotypic resistance to the corresponding antibiotics. Additionally, 6/76 (7.9%) isolates carried *vanA* and *vanC* simultaneously, while 7/76 (9.2%) isolates harboured *ermA* and *ermC* in combination. Furthermore, 8/76 (10.5%) of the isolates carried both *mecA* and *mecC*.

### SCC*mec* typing of MRSA isolates

**Figure 2:**
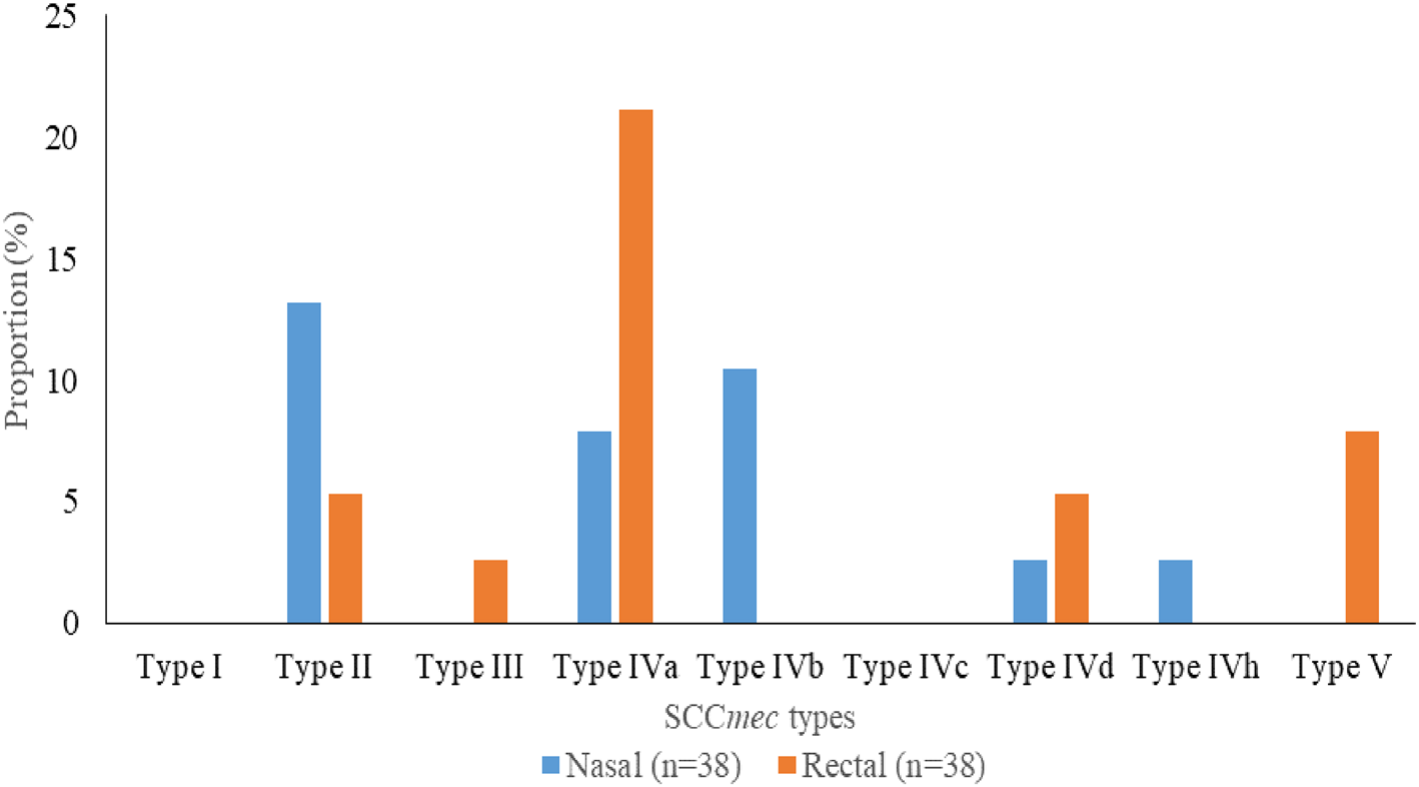
Distribution of SCC*mec* gene types in the staphylococcal isolates.

*SCCmec* typing of MRSA includes: Type II 7/76 (9.2%), Type III one (1.3%), Type IVa 11 (14.5%), Type IVb four (5.3%), Type IVd three (3.9%), Type IVh one (1.3%), Type V three (3.9%). None of the isolates amplified genes for *SCCmec* Type I and IVc (Fig. 2). All *SCCmec*-positive isolates harboured the *PVL* determinant.

### The biofilm formation profile of MRSA isolates

**Figure 3:**
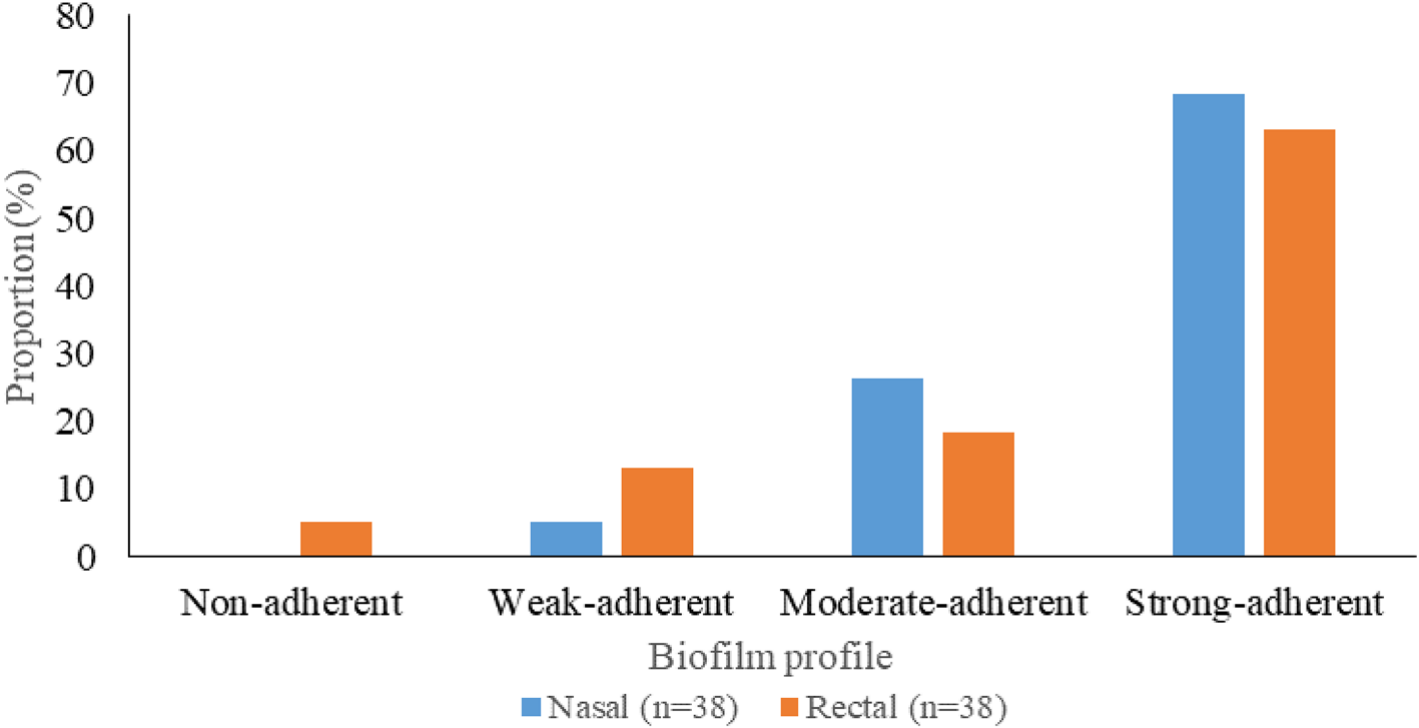
Distribution of biofilm formation in the staphylococcal isolates.

The biofilm-forming capacity of the nasal isolates indicates that none of the isolates was non-adherent; 2 (5.3%) were weak-adherent, 10(26.3%) were moderate-adherent, and 26(68.4%) were strong-adherent. Similarly, the biofilm-forming capacity of the rectal isolates shows that 2(5.3%) were non-adherent, 5(13.2%) were weak-adherent, 7(18.4%) were moderate-adherent, and 24(63.2%) were strong-adherent (Fig. 3). Overall, the distribution of biofilm formers includes no formers 2/76(2.6%), weak formers 7/76(9.2%), moderate formers 17/76(22.4%), and strong formers 50/76(65.8%). It is noteworthy that all isolates with *icaA* and *icaB* genes formed moderate to strong biofilms.

## Discussion

AMR, inherent but exacerbated by excessive use, poses a global threat. Nigeria's Ministry of Agriculture warns of its impact on food security and global health^19^. Our study's *S. aureus* rates align with those from Denmark, while higher rates are noted from studies in Norway and the USA and lower rates from studies in Spain, South Africa, and Tunisia^21,22^. Our MRSA prevalence mirrors those from Latvia, contrasting lower rates from those in Portugal, Indonesia, Mexico, Nigeria, and Korea^1,11^. Elevated *S. aureus* and MRSA prevalence in our pastoral-focused study suggest influences from unmonitored feeds, water exposure, varied environmental factors, treatment, breeding systems, sanitation, seasons, location, and animal species^21^. Intensifying surveillance of AMR in animal populations, particularly those integral to the food supply, is critical, especially in developing nations with limited epidemiological data. Our research identifies MDR MRSA, reinforcing our earlier findings^13,14^. These strains pose risks to both animal and human health through products from colonized animals, aligning with studies highlighting food-producing animals as significant AMR MRSA reservoirs. The potential circulation of these strains between animals and humans is substantial^6^. Penicillin resistance (100%) in our study mirrors findings in Nigeria, Bangladesh, and Ethiopia, while lower rates (72.6–98.5%) were reported in India, Ethiopia, and China^23,24^.

In contrast to our findings, a study reported high sensitivity (96%) of S. aureus of cattle origin to cefotaxime and ceftaroline^25^. However, consistent with our research, another study revealed universal resistance of *S. aureus* isolates to cefotaxime^26^. This emphasizes the crucial role of veterinarians in judicious antibiotic use for food-producing animals, advocating for prescription-based administration to treat infections^19^. Contrary to our study, Naas et al.^26^ reported higher clindamycin resistance, and Kou et al.^24^ found MRSA more sensitive to clindamycin. The inefficacy of penicillins, cephalosporins, and carbapenems against *S. aureus* has been noted globally, linked to their extensive use in veterinary medicine^4^. Resistance patterns vary regionally and are closely tied to levels of antimicrobial usage (AMU). LA- *S. aureus* strains, according to Omwenga et al.^4^, primarily resist tetracycline and ampicillin. Our study identified significant tetracycline resistance, although lower than Onyenwe et al.^25^ and Gali et al.^23^, but higher than Kou et al.^24^. Fluoroquinolone resistance aligns with previous research^11,27^. MRSA isolates in our study moderately responded to kanamycin, gentamicin, and vancomycin, consistent with Onyenwe et al.^25^ and Kou et al.^24^ but not Naas et al.^26^. Vancomycin's comparatively higher effectiveness might stem from more judicious use in the study area^28^. Bernier-Lachance et al.^3^ found sensitivity to various antibiotics, with only certain classes showing no resistance in our study.

In contrast to Silva et al.^11^, our MRSA isolates did not universally resist penicillin, ciprofloxacin, clindamycin, and erythromycin. Similar to Back et al.^1^, MRSA strains in our study resisted ampicillin but were sensitive to teicoplanin. Variable resistance rates are linked to geographic differences in AMU^4^. Our investigation revealed a MAR index ranging from 0.15 to 70, surpassing the 0.2 threshold, indicating inappropriate antibiotic use and posing risks to public health^29^.

The excessive use of antibiotics in livestock, observed in our study area, may be a key driver in the selection of AMR strains. The diverse resistance patterns identified in our research can be attributed to the prolonged and indiscriminate application of various antimicrobial classes in modern agriculture, as highlighted in previous studies^27,30^. Bernier-Lachance et al.^3^ findings, where all isolates exhibited intermediate or full resistance to β-lactams, align with our results. While our study reported lower levels of MDR compared to other research^3,11^, Omwenga et al.^4^ identified 94% of MDR *S. aureus* isolates as MRSA, inherently resistant to commonly prescribed antibiotics. Back et al.^1^ proposed that the co-selection pressure linked to the MDR phenotype significantly contributed to the emergence and continuity of LA-MRSA. The surge in MDR MRSA is linked to extensive antibiotic use in veterinary and human healthcare^4^. Genetic factors carried by resistant isolates contribute to observed resistance patterns, highlighting the pivotal role of selective pressure from antibiotics^31,32^. The dissemination of AMR in *S. aureus* involves various MGEs^14^. Recognizing the genetic factors linked to resistance phenotypes is essential for comprehending the molecular mechanisms underlying the rise and dissemination of AMR. The genetic profiles of resistance phenotypes closely align with resistant isolates, suggesting that genes carried by LA-MRSA strains may underpin observed resistance phenotypes.

In this study, the identification of the *mecA* gene in all LA-MRSA isolates, as reported in the Silva et al.^11^ investigation from Quails in Portugal as well Gaddafi et al.^33^ from dairy cows in Nigeria, indicates its consistent presence and remains the definitive indicator for MRSA detection. *mecA*-positive staphylococcal strains, immune to all β-lactam antibiotics, highlight the significance of this genetic marker^34^. Using PCR for MRSA detection mitigates the risk of false-negative results common with phenotypic methods. Instances of *mecA*-positive *S. aureus* strains with phenotypic oxacillin susceptibility have been documented, emphasizing the need for a combined approach evaluating both phenotype and genotype to prevent false outcomes in MRSA identification^35^. Our study revealed that 81.6% of MRSA isolates were categorized as community-acquired MRSA (CA-MRSA) due to the presence of *PVL* genes. This rate surpasses previous findings^36^ and falls below others^3^. *PVL* genes, virulence factors associated with CA-MRSA, enhance virulence and serve as genetic markers, differentiating them from hospital-associated MRSA^37^. Notably, *PVL* and human-related immune evasion cluster genes were absent in ST398 LA-MRSA strains in previous studies^7,11^. The acquisition of *PVL* and *TSST-1* genes in LA-MRSA poses a considerable threat to public health. Bernier-Lachance et al.^3^ reported negative results for *tsst-1* alleles, contrasting with our findings.

Apart from the *mecA* gene, the MRSA isolates in our study contained genes associated with resistance to tetracyclines, lincosamides, and macrolides, consistent with findings in previous research^20^. The presence of these genes aligns with observed phenotypic AMR. Correlations were identified, such as tetracycline use with *tetM* detection, penicillins with *mecA/mecC*, vancomycin with *vanA* and *vanC*, and macrolides with *ermA* and *ermC*, as reported elsewhere^4,11^. The occurrence of MDR *S. aureus* and *tetM* detection has been linked to AMU^4^. Our study detected *ermA* in proportions comparable to studies in Kenya but lower than in Egypt, with *erm* genes facilitating adjustments to 23S rRNA^38^. The administration of tylosin might influence resistance to macrolides for treating prevalent diseases in the region. Similar to Silva et al.^11^, our study identified macrolide-lincosamide resistance genes, including *ermC* or a combination of *ermB* and *ermC*. Tetracycline resistance genes, including *tetM*, were found, which is consistent with Silva et al.^11^. Tetracycline and penicillin, frequently used in livestock farming, contribute to the prevalence of these genes^20^. The *tetM* genes are typically located in transposons or chromosomes^39^. Resistance determinants often cluster on MGEs, intensifying co-selection and resulting in co-resistance to other antibiotics. Plasmids carrying tetracycline-resistant genes may also harbour supplementary genes, and the extensive utilization of antibiotics with broad-spectrum activity could contribute to the co-selection of resistance genes.

A previous study^9^ reported a higher prevalence of *SCCmec* gene cassette type V compared to type I among LA-MRSA strains, diverging from our findings. A study from Greece identified *SCCmec* type V elements in both human and LA-MRSA isolates^7^. The predominant *SCCmec* type V in CC398 LA-MRSA typically carries genes detoxifying heavy metals like cadmium and copper, commonly used in farm growth promotion^40^. Copper-resistant genes contribute to bacterial survival^41^. ST398 isolates contained *SCCmec* type V, while ST6831 was untypeable^11^. ST398 and ST541 MRSA isolates, except for two non-typeable LA-MRSA, carried *SCCmec* type V^1^. Other MRSA isolates with non-CC398 genotypes had *SCCmec* IV or *SCCmec* II. Although MLST was not conducted, certain isolates in our study contained *SCCmec* types II, III, IVa, IVb, IVd, IVh, and V. Isolates with *SCCmec* V also proportionally carried *tetK*^42^. The co-localization of *czrC* and *mecA* genes in *SCCmec* V suggests zinc inclusion in livestock feed might promote *czr* in CC398 LA-MRSA^1^.

*S. aureus*'s biofilm-forming capability, a crucial virulence factor, intensifies AMR, posing formidable challenges in infection management^2,43^. Studies highlight that biofilm-forming strains exhibit elevated MDR and methicillin resistance compared to non-biofilm strains^44^. Biofilms, with their adherence to diverse surfaces, enhance antibiotic resistance and survival in varied environments^29,45,46^. In contrast, Bernier-Lachance et al.^3^ found that all isolates formed biofilms, with biofilm-forming isolates possessing *icaACD* genes, which are essential for biofilm formation. In our study, some isolates formed biofilms carrying only *icaA* and *icaB* genes. Positive MSCRAMMs gene probes in MRSA indicate their genetic capability for efficient adhesion, promoting biofilm formation, potentially enhancing persistence colonization, and facilitating zoonotic transmission^3^. Key limitations of the study include limited sampling diversity as it did not include humans and the environment, lack of investigation into transmission pathways involving potential LA-MRSA transmission between livestock and humans or other environmental reservoirs, limited exploration of antimicrobial resistance mechanisms with exploring the resistance evolution and transmission dynamics, and reliance on conventional methods such as PCR techniques, without exploring complementary approaches, such as whole-genome sequencing.

## Conclusion

Detecting MDR MRSA in livestock raises health concerns for handlers and the community. Improving hygiene around food-producing animals is crucial to mitigate microbiological risks. Many isolates in our study showed heightened AMR and robust biofilm-forming abilities. Routine monitoring of biofilm development and AMR in *S. aureus* is essential for effective treatment and AMR control. Implementing immunizations, proper animal care, and biosecurity measures can reduce infections and antibiotic use in livestock. Governments and policymakers must lead in establishing sustainable policies to curb indiscriminate antibiotic use, promoting both human and animal health.

## Materials and methods

### Study area and sample collection

Samples were obtained from free-range cows in Edo State. The farmers commonly administered tetracyclines and penicillin as the predominant antibiotics for livestock. Other antibiotics, such as fluoroquinolones, sulphonamides, cephalosporins and streptomycin, are also used. Single (oral, topical or parenteral) preparations of the antibiotics were mostly observed during field analysis, followed by > 1 preparation, while a combination of preparations of antibiotics was less commonly observed. The animal handlers usually source antibiotics from veterinary pharmacy shops, veterinary clinics, market displays, and drug hawkers. Non-antibiotic drugs such as multivitamins, anthelminthic drugs, traditional concoctions, salts, ashes, pepper, onion, potash and herbs were also observed to be used. Information gathered revealed that the antibiotics used were recommendations by veterinarians, from personal experience, on recommendations by drug sellers, on advice from fellow farmers, or based on advertisements.

The study adopts a longitudinal design, wherein animals serve as their controls. The sampling procedures adhered to the guidelines set by the European Food Safety Authority^47^. The sample size for this study was determined using the formula:

$$Sample \left(N\right)=\frac{{({Z}_{1-\propto /2})}^{2} P(1-P) }{{d}^{2}}$$

where: *Z*_*1-α/2*_ = Standard normal variant at 5% type I error (P < 0.05), *P* = Expected prevalence based on previous studies (ranging from 3.4% to 35.7%)^1,22–24,28,29,36^, *d* = Absolute error or precision (set at 5%).

Based on this calculation, the expected maximum number of samples was 353. However, a total of 400 swab samples, including 200 rectal and 200 nasal samples, were systematically collected from free-range cows in Edo State, Nigeria, from March 2018 to February 2019. The rectal and nasal samples were taken from the same cows. The swab samples were acquired from the cows using sterile swab sticks moistened with normal saline, ensuring a well-organized approach to prevent any duplication of samples. Subsequently, the collected samples were promptly transported to the laboratory of the Applied Microbial Processes and Environmental Health Research Group (AMPEHRG) at the University of Benin, located in Benin City, Nigeria. The samples were kept on ice during transport to facilitate the subsequent processing. In addition to the sample collection, information regarding the treatment history and the types of antibiotics used was collected to provide a comprehensive description of the study population.

### Ethics declaration

All methods described in this study were conducted following the guidelines and regulations outlined by relevant institutional and national committees for research involving animals and human subjects. The research protocols were reviewed and approved by the Research and Ethics Committee, State Ministry of Health, Edo State, Nigeria, with reference number Ha.737/5/T1/019 before the commencement of the study. Additionally, we confirm that all methods reported in this manuscript adhere to the guidelines provided by the ARRIVE (Animal Research: Reporting of In Vivo Experiments) guidelines (https://arriveguidelines.org) for reporting experiments involving animals.

### Laboratory identification of *S. aureus* and MRSA

The swab sticks were introduced into tryptone soy broth (Merck, Darmstadt, Germany) and subsequently placed in an incubator at 37 °C for a duration of 18 to 24 h. Following incubation, the broth was subjected to a streaking technique on both Baird-Parker agar (Merck, Darmstadt, Germany) and MRSA selective agar plates (CHROMagarTM MRSA-ITK Diagnostics BV, Netherlands). These plates were then incubated at 37 °C for 24 h. Any colonies exhibiting circular, smooth, convex, moist, grey to jet-black characteristics on Baird-Parker agar, as well as rose to mauve colonies on MRSA selective agar plates, were considered as presumptive *S. aureus* and presumptive MRSA, respectively. To further confirm the identity of these colonies, one colony from each plate was isolated and purified by cultivation in nutrient agar (Lab M, Lancashire, United Kingdom). These colonies were then subjected to another 18-h incubation at 37 °C and subsequently stored at 4 °C on nutrient agar slants. The identification of the isolates was based on a series of cultural, morphological, and biochemical tests, including Gram staining, 3% potassium hydroxide testing, coagulase, catalase, and anaerobic utilization of mannitol and glucose, following the procedures outlined by Tallent et al.^48^. Only the MRSA isolates underwent further characterization. A positive control in the form of *S. aureus* (ATCC 12600) was employed for reference purposes.

### DNA extraction and molecular characterization of the MRSA isolates

DNA extraction was conducted following a previously established protocol^30,49^. To resuscitate all isolates, they were placed in 5 mL of tryptone soy broth after an initial 24-h incubation at 37 °C. Following this, cells were collected by centrifuging 2 mL of the incubated broth in sterile Eppendorf tubes at 5,000 rpm for 10 min. The cell deposit underwent a washing step with normal saline after discarding the supernatant, followed by re-centrifugation at 5000 *rpm* for 3 min. The cell pellet was subsequently re-suspended in a microcentrifuge tube containing a rapid lysis buffer. This lysis buffer included the following components: 100 mM NaCl, 10 mM Tris–HCl at pH 8.3, 1 mM EDTA at pH 9.0, and 1% Triton X-100. The mixture was boiled for 15 min, and subsequently, it was centrifuged at 10,000 rpm. The resulting supernatant was collected and stored at − 20 °C for future use as the template DNA. To identify *S. aureus* (based on the *nuc* gene), a polymerase chain reaction (PCR) was conducted using specific primers, as previously reported by Brakstad et al.^50^, with *S. aureus* ATCC 12600 serving as the positive control. The specific primer used for identification is detailed in Supplementary Table 1 for the amplification of AMR genes, such as methicillin resistance (*mecA*) that was also used as a determinant to confirm the presumptive MRSA isolates molecularly, tetracycline (*tetM*), erythromycins (*ermA*, *ermC*), vancomycin (*vanA*, *vanC*), as well as virulence genes including PVL, *tsst*-1, intercellular adhesion proteins (*icaA* and *icaB*), procedures outlined in prior studies^51–53^ were followed. *SCCmec* typing of MRSA isolates involved PCR amplification of *SCCmec* types 1 to V and subtype *SCCmec* (Iva to d) as described by Okuma et al.^54^, Ma et al.^55^, and Zhang et al.^56^. Specific primer sets listed in Supplementary Table 1 were used. The PCR products were electrophoresed on a 1% agarose gel at 110 V for 45 min and visualized after ethidium bromide staining using a transilluminator (Vilber Lourmat, EBOX VX5, France).

### The biofilm formation profile of MRSA isolates

After the molecular confirmation of the MRSA isolates, pure MRSA colonies were introduced into 4.5 mL of tryptone soy broth (TSB) and then subjected to incubation at 37 °C for 18 h. Subsequently, these cultures underwent centrifugation for 2 min at 12,000 *rpm*. The resulting cell pellets were washed and re-suspended in phosphate-buffered solution (PBS) adjusted to pH 7.2, targeting 0.5 McFarland standards. The prepared suspension inoculants were introduced into the wells of sterile 96-well polystyrene microtiter plates containing 20 mL of cell suspensions and 180 mL of TSB to investigate the adherence of Staphylococci to a solid surface, following the procedure described previously^30^. The negative control wells contained only TSB broth, while *S. aureus* ATCC 12600 served as the positive control. Each of the assays was performed in triplicate to calculate the mean value. Based on previously established protocols^30^, biofilm formation was classified as non-producing/negative (ODi < ODc), weak/poor-producing (ODc < ODi < 0.1), moderate/intermediate-producing (0.1 < ODi < 0.12), or strong producer (ODi > 0.12).

### Antimicrobial susceptibility profile of the MRSA isolates

Antimicrobial susceptibility testing was conducted through the Kirby-Bauer disk diffusion method. To do this, a suspension of the test isolates was prepared at a 0.5 McFarland standard. Aseptically, a suspension of the isolated test strains was streaked on Mueller–Hinton agar (obtained from Lab M, Lancashire, UK), and the corresponding antibiotic discs were aseptically positioned on the inoculated agar. The antibiotic discs used in this study were all sourced from Mast Diagnostics, United Kingdom. The disc employed includes penicillin G (PEN) (10 units), piperacillin (PTZ) (100 µg), gentamicin (GEN) (10 µg), kanamycin (KAN) (30 µg), tetracycline (TET) (30 µg), imipenem (IMP) (10 µg), meropenem (MEM) (10 µg), ertapenem (ETP) (10 µg), ceftaroline (CRO) (30 µg), cefotaxime (CTX) (30 µg), erythromycin (ERY) (15 µg), clindamycin (CLI) (30 µg), ciprofloxacin (CIP) (10 µg), quinupristin-dalfopristin (QDA) (15 µg), and linezolid (LIZ) (30 µg). The agar plates were allowed to air-dry at room temperature for approximately 10 min, followed by an incubation at 37 °C for 24 h. The diameter of the inhibition zone was measured using a transparent meter rule and interpreted according to recognized criteria, categorizing the strains as sensitive (S), intermediate resistant (I), or resistant (R) in accordance with the standards recommended by the Clinical Laboratory Standards Institute^57^. The minimum inhibitory concentration (MIC) protocol was carried out by preparing stock solutions of each of the antibiotics at various concentrations (vancomycin 1–32 µg/mL, oritavancin 0.12–0.50 µg/mL, teicoplanin 4–64 µg/mL, daptomycin 0.5–4 µg/mL, and tedizolid 0.25–4 µg/mL), typically using serial dilutions. The standardized bacterial suspension was inoculated onto Mueller–Hinton broth (obtained from Lab M, Lancashire, UK). The antibiotic solutions were added to the bacterial cultures, ensuring that each well on the 96 well microtiter plate contained a different concentration of the antibiotic being tested. The broth cultures were incubated at 37 °C for 24 h. After incubation, bacterial growth was assessed in each well. The MIC is defined as the lowest concentration of antibiotic that completely inhibits visible bacterial growth. The MIC values obtained were compared with established interpretive guidelines and MIC breakpoints provided by the CLSI^57^. This helped to determine whether the bacteria were susceptible, intermediate, or resistant to the antibiotics being tested.. *S. aureus* ATCC 12600 was used as a positive control in this experiment.

### Statistical analysis

All data in this research were subjected to analysis utilizing the statistical package SPSS version 21.0 and Microsoft Excel 2013. Descriptive statistics, which included the calculation of means and standard deviations, were employed for data summarization. A One-Way Analysis of Variance (ANOVA) was employed to analyze multiple variables, with the Duncan Multiple Range test used to identify significant differences between means. A probability value below 0.05 was regarded as indicative of statistical significance.

### Supplementary Information

Supplementary Information.

## Data Availability

The datasets generated and analyzed during the current study are available in the manuscript. All relevant data supporting the findings of this study are included in the supplementary information files. Any other information can be obtained from the corresponding author upon reasonable request.
