# Supplementary material for: Characterization of resistance and virulence factors in livestock-associated methicillin-resistant Staphylococcus aureus
Source: Sci Rep. 2024 Jun 9;14:13235. doi: 10.1038/s41598-024-63963-3 (PMC11163002; doi:10.1038/s41598-024-63963-3)
Supplement: Supplementary file 1 — Supplementary Information. [file 41598_2024_63963_MOESM1_ESM.docx]

**Characterizing Livestock-Associated Methicillin-Resistant *Staphylococcus aureus* (LA-MRSA): Resistance, and Virulence Factors**

Abeni Beshiru^1,2^, Isoken H. Igbinosa^1,3^, Olajide Akinnibosun^1,4^, Abraham G. Ogofure^1^, Afamefuna Dunkwu-Okafor^1^, Kate E. Uwhuba^2^, Etinosa O. Igbinosa^1,^*

^1^Applied Microbial Processes & Environmental Health Research Group, Faculty of Life Sciences, University of Benin, PMB 1154, Benin City 300283, Nigeria.

^2^Department of Microbiology, College of Natural and Applied Sciences, Western Delta University, Oghara, Nigeria.

^3^Department of Environmental Management & Toxicology, Faculty of Life Sciences, University of Benin, PMB 1154, Benin City 300283, Nigeria.

^4^Department of Microbiology, Faculty of Science, Federal University of Health Sciences, Otukpo, PMB 145, Otukpo 927101, Nigeria.

*Corresponding author Email: EOI- Etinosa.Igbinosa@uniben.edu

**Supplementary Table 1**. Primers used in the study

| **Primer** | **Target genes** | **Primer sequences ( 5' →3')** | **Size (bp)** | **References** |
| --- | --- | --- | --- | --- |
| *Staphylococcus aureus* | *nuc* | F:GCGATTGATGGTGATACGGTT  R: AGCCAAGCCTTGACGAACTA AAGC | 270 | Brakstad et al.^1^ |
| Panton valentine leukocidin | *Pvl* | F:ATCATTAGGTAAAATGTCTGGACATGATCCA R:GCATCAAGTGTATTGGATAGCAAAAGC | 433 | McClure et al.^2^ |
| Toxic shock syndrome toxin 1 | *tsst*-1 | F:GCTTGCGACAACTGCTACAG  R: TGGATCCGTCATTCATTGTTAT | 559 | Monday et al.^3^ |
| Intercellular adhesion protein B | *ica*B | F:CTGATCAAGAATTTAAATCACAAA  R:AAAGTCCCATAAGCCTGTTT | 302 | Arciola et al.^4^ |
| Intercellular adhesion protein A | *ica*A | F:ACAGTCGCTACGAAAAGAAA  R:GGAAATGCCATAATGACAAC | 103 | Arciola et al.^4^ |
| Methicillin resistance | *mec*A | F:AAAATCGATGGTAAAGGTTGGC  R: AGTTCTGCAGTACCGGATTTGC | 532 | Strommenger et al.^5^ |
| Methicillin resistance | *mec*C | F:TGAACGAAGCAACAGTACACC  R:AGATCTTTTCCGTTTTCAGCCT | 238 | Ciesielczuk et al.^6^ |
| Tetracyclines | *tet*M | F:GTGGACAAAGGTACAACGAG  R:CGGTAAAGT TCG TCACACAC | 406 | Ng et al.^7^ |
| Erythromycins | *erm*A | F: TATCTTATCGTTGAGAAGGGATT  R:CTACACTTGGCTTAGGATGAAA | 139 | Martineau et al.^8^ |
| Erythromycins | *erm*C | F:CTTGTTGATCACGATAATTTCC  R: ATCTTTTAGCAAACCCGTATTC | 190 | Martineau et al.^8^ |
| Vancomycin | *vanA* | F:GCGCGGTCCACTTGTAGATA  R: TGAGCAACCCCCAAACAGTA | 314 | Nam et al.^9^ |
| Vancomycin | *vanC* | F: ATCCAAGCTATTGACCCGCT  R: TGTGGCAGGATCGTTTTCAT | 402 | Nam et al.^9^ |
| Type I | *ORF E008* | F:GCTTTAAAGAGTGTCGTTACAGG  R: GTTCTCTCATAGTATGACGTCC | 613 | Zhang et al.^10^ |
| Type II | *kdpE* | F:GATTACTTCAGAACCAGGTCAT  R: TAAACTGTGTCACACGATCCAT | 287 | Kondo et al.^11^ |
| Type III | *J1 III* | F:CATTTGTGAAACACAGTACG  R:GTTATTGAGACTCCTAAAGC | 243 | Milheirico et al.^12^ |
| Type Iva | *ORF CQ002* | F: GCCTTATTCGAAGAAACCG  R:CTACTCTTCTGAAAAGCGTCG | 776 | Zhang et al.^10^ |
| Type IVb | *J1* Ivb | F:AGTACATTTTATCTTTGCGTA  R:AGTCATCTTCAATATGGAGAAAGTA | 1000 | Okuma et al.^13^ |
| Type IVc | *Ivc* | F:TCTATTCAATCGTTCTCGTATT  R:TCGTTGTCATTTAATTCTGAACT | 677 | Ma et al.^14^ |
| Type IVd | *CD002* | F:AATTCACCCGTACCTGAGAA  R:AGAATGTGGTTATAAGATAGCTA | 1242 | Kondo et al.^11^ |
| Type IVh | *J1* | F:TTCCTCGTTTTTTCTGAACG  R:CAAACACTGATATTGTGTCG | 663 | Milheirico et al.^12^ |
| Type V | *ORF V011* | F:GAACATTGTTACTTAAATGAGCG  R:TGAAAGTTGTACCCTTGACACC | 325 | Zhang et al.^10^ |

**References**

1. Brakstad, O. G., Aasbakk, K. & Maeland, J. A. Detection of *Staphylococcus aureus* by polymerase chain reaction amplification of the *nuc* gene. *J. Clin. Microbiol*. **30**, 1654-1660 (1992).
2. McClure, J. A. *et al*. Novel multiplex PCR assay for detection of the staphylococcal virulence marker panton-valentine leukocidin genes and simultaneous discrimination of methicillin-susceptible from -resistant staphylococci. *J. Clin. Microbiol*. **44**, 1141-1144 (2006).
3. Monday, S. R. & Bohach, G. A. Use of multiplex PCR to detect classical and newly described pyrogenic toxin genes in staphylococcal isolates. *J. Clin. Microbiol*. **37**, 3411-3414 (1999).
4. Arciola, C. R. *et al*. A multiplex PCR method for the detection of all five individual genes of *ica* locus in *Staphylococcus epidermidis*. A survey on 400 clinical isolates from prosthesis-associated infections. *J. Biomed. Mater. Res. A* **75**, 408–413 (2005).
5. Strommenger, B., Kettlitz, C., Werner, G. & Witte, W. Multiplex PCR assay for simultaneous detection of nine clinically relevant antibiotic resistance genes in *Staphylococcus aureus*. J. Clin. Microbiol. **41**, 4089-4094 (2003).
6. Ciesielczuk, H., Xenophontos, M. & Lambourne, J. Methicillin-resistant *Staphylococcus aureus* harboring *mecC* still eludes us in East London, United Kingdom. *J. Clin. Microbiol*. **57**, e00020-19 (2019).
7. Ng, L. K., Martin, I., Alfa, M. & Mulvey M. Multiplex PCR for the detection of tetracycline-resistant genes. *Mol. Cell. Probes* **15**, 209–215 (2001).
8. Martineau, F. *et al*. Correlation between the resistance genotype determined by multiplex PCR assays and the antibiotic susceptibility patterns of *Staphylococcus aureus* and *Staphylococcus epidermidis*. *Antimicrob. Agents Chemother*. **44**, 231–238 (2000).
9. Nam, S., Kim, M. J., Park, C., Park, J. G. & Lee, G. C. Detection and genotyping of vancomycin-resistant *Enterococcus* spp. by multiplex polymerase chain reaction in Korean aquatic environmental samples. *Int. J. Hyg. Environ. Health* **216**, 421–427 (2013).
10. Zhang, K., McClure, J. A., Elsayed, S., Louie, T. & Conly, J. M. Novel multiplex PCR assay for characterization and concomitant subtyping of *Staphylococcus* cassette chromosome mec types I to V in methicillin-resistant *Staphylococcus aureus*. *J. Clin. Microbiol*. **43**, 5026–5033 (2005).
11. Kondo, Y. *et al*. Combination of multiplex PCRs for staphylococcal cassette chromosome mec type assignment: rapid identification system for *mec*, *ccr*, and major differences in junkyard regions. Antimicrob. Agents Chemother. **51**, 264–274 (2007).
12. Milheirico, C., Oliveira, D. C. & de Lencastre, H. Update to the multiplex PCR strategy for assignment of mec element types in *Staphylococcus aureus*. *Antimicrob. Agents Chemother*. 51, 3374–3377 (2007).
13. Okuma, K. *et al*. Dissemination of new methicillin-resistant *Staphylococcus aureus* clones in the community. *J. Clin. Microbiol*. **40**, 4289–4294 (2002).
14. Ma, X. X. *et al*. Community-acquired methicillin-resistant *Staphylococcus aureus*, Uruguay. *Emerg. Infect. Dis*. **11**, 973–976 (2005).
